# Use of External Fixators as a 3-Dimensional Navigation Drill Guide for Arthroscopic Ankle Arthrodesis

**DOI:** 10.1155/2020/6072143

**Published:** 2020-11-06

**Authors:** Young Uk Park, Hyong Nyun Kim

**Affiliations:** ^1^Department of Orthopedic Surgery, Ajou University Hospital, Ajou University School of Medicine, Suwon, Gyeonggi-do, Republic of Korea; ^2^Department of Orthopedic Surgery, Kangnam Sacred Heart Hospital, Hallym University College of Medicine, Seoul, Republic of Korea

## Abstract

In this article, we describe a novel technique using external fixators and cannulated screws to construct a 3-dimensional navigation drill guide to predict the screw trajectory before screw insertion that can prevent screw collision during arthroscopic ankle arthrodesis. Four orthopedic residents who had no prior experience of ankle arthrodesis were instructed on how to use the 3-dimensional navigation drill guide and where to insert the screws for ankle arthrodesis. Each resident inserted 6.5 cannulated screws on 8 sawbone ankle models using the device and the C-arm fluoroscopy. An experienced attending surgeon also inserted the same screws on 2 sawbone ankle models to find out if there is any difference between the experienced and inexperienced surgeons. All four residents and an attending surgeon did not experience any collision of screws for the three cannulated screws. Notably, one resident had collision of the 4th screw on his first sawbone model. On the second saw bone model, all surgeons could insert 5 screws without redrilling. A 3-dimensional navigation drill guide constructed with external fixators can assist surgeons in implementing percutaneous screws for arthroscopic ankle arthrodesis.

## 1. Introduction

**Figure 1:**
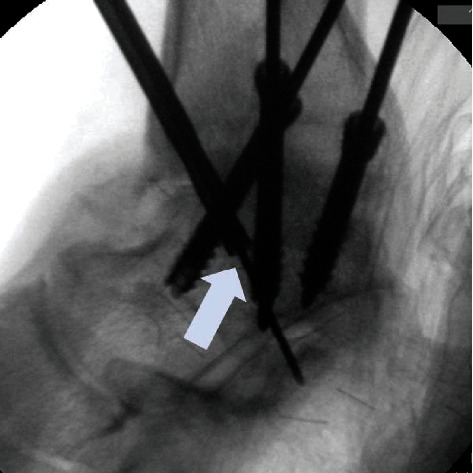
A large diameter drill bit collided (arrow) with the screws inside the talus.

Traditionally, ankle arthrodesis has been the preferred surgical method to treat end-stage ankle arthritis [1, 2]. Recently, arthroscopic arthrodesis has gained popularity because of the numerous advantages it offers over open arthrodesis including minimal postoperative pain and wound problems, shorter operative time, minimal blood loss, and shorter hospital stay [3–8]. Several studies have shown high rates of union comparable to open arthrodesis. It has been theorized that the arthroscopic technique with minimal dissection protects the periarticular blood supply, which probably enhances the fusion process and facilitates a more rapid repair. However, it is not without nonunion [9, 10]. Crosby et al. [10] reported 26% of radiologic nonunion in 42 patients with arthroscopic ankle arthrodesis. In arthroscopic ankle arthrodesis, the ankle is fixed with two or three screws percutaneously [11–13]. Screw fixation is minimally invasive; however, it is not rigid that the number of screws can influence the initial stability of the fusion [14–17]. It is suggested that a large number of screws provide a more rigid fixation [14, 17]. However, compared to the open technique, the insertion of screws under C-arm fluoroscopic guidance for arthroscopic arthrodesis is cumbersome as the joint anatomy is not opened and visualized. There is a possibility that the screws with large diameters could collide inside the talus (Figure 1).

When the drill bit with the large diameter collides with the screws inside the talus, it may not be possible to advance the drill bit across the screws. Giving up the drilled hole and attempting a new trajectory will leave a large hole which may decrease the stability of the ankle arthrodesis. Using cannulated screws and guide wires cannot be a solution as the guide wire is much smaller in diameter; it has the limitation of predicting screw collisions inside the talus. When the screw trajectory can be predicted and analyzed prior to the screw insertion, it will help surgeons to prevent the drilling of unused holes and save operation time and lower radiation exposure under C-arm fluoroscopy. In this article, we describe a novel technique using external fixators and cannulated screws to construct a 3-dimensional navigation drill guide to predict the screw trajectory before screw insertion that can prevent screw collision.

## 2. Materials and Methods

### 2.1. Preparation of a 3-Dimensional Navigation Drill Guide

**Figure 2:**
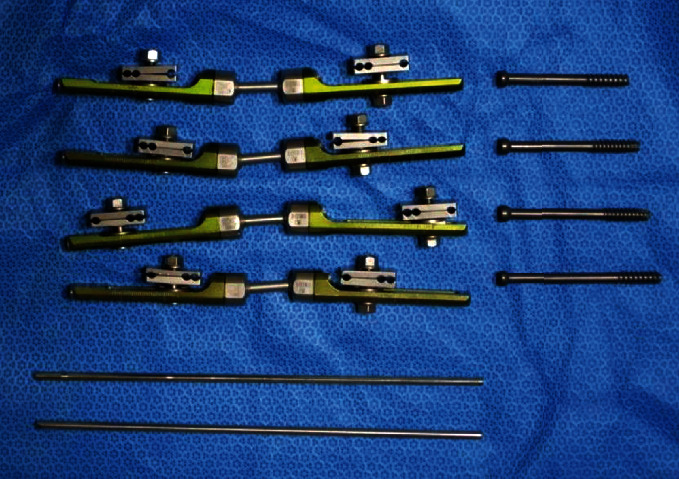
A 3-dimensional navigation drill guide is constructed using external fixators used for fixation of wrist fracture, 5.0 cannulated screws, and large diameter S-pins.

Prior to the surgery, a 3-dimensional navigation drill guide is constructed using external fixators used for the fixation of wrist fracture, 5.0 cannulated screws, and large diameter S-pins (Figure 2).

**Figure 3:**
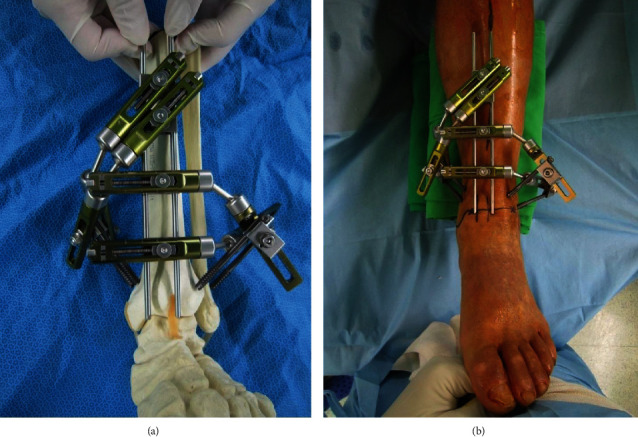
(a) The two large diameter S-pins are used to form the main frame of the device. The external fixators are connected to the S-pins by the pin clamps where they are appropriate to guide screw fixation. Cannulated screws attached to the external fixators by the pin clamps are used as the drill guide where guide pins can be inserted through the cannulated hole of the screws. (b) The 3-dimensional navigation drill guide is positioned on the ankle joint where the lower-end of the medial S-pin will sit on the medial corner of the ankle joint.

The two large diameter S-pins are used to form the main frame of the device. The external fixators are connected to the S-pins by the pin clamps where they aid in guiding during the screw fixation. Cannulated screws attached to the external fixators by the pin clamps are used as the drill guide where guide pins can be inserted through the cannulated hole of the screws (Figure 3(a)). Joints of the external fixators are adjusted to decide the trajectory of the cannulated screws. The device is constructed in two different sides (right and left), and once constructed, they can be reused with minor adjustment for all cases of arthroscopic ankle arthrodesis. The 3-dimensional navigation drill guide is sterilized before surgery.

### 2.2. Operative Technique

**Figure 4:**
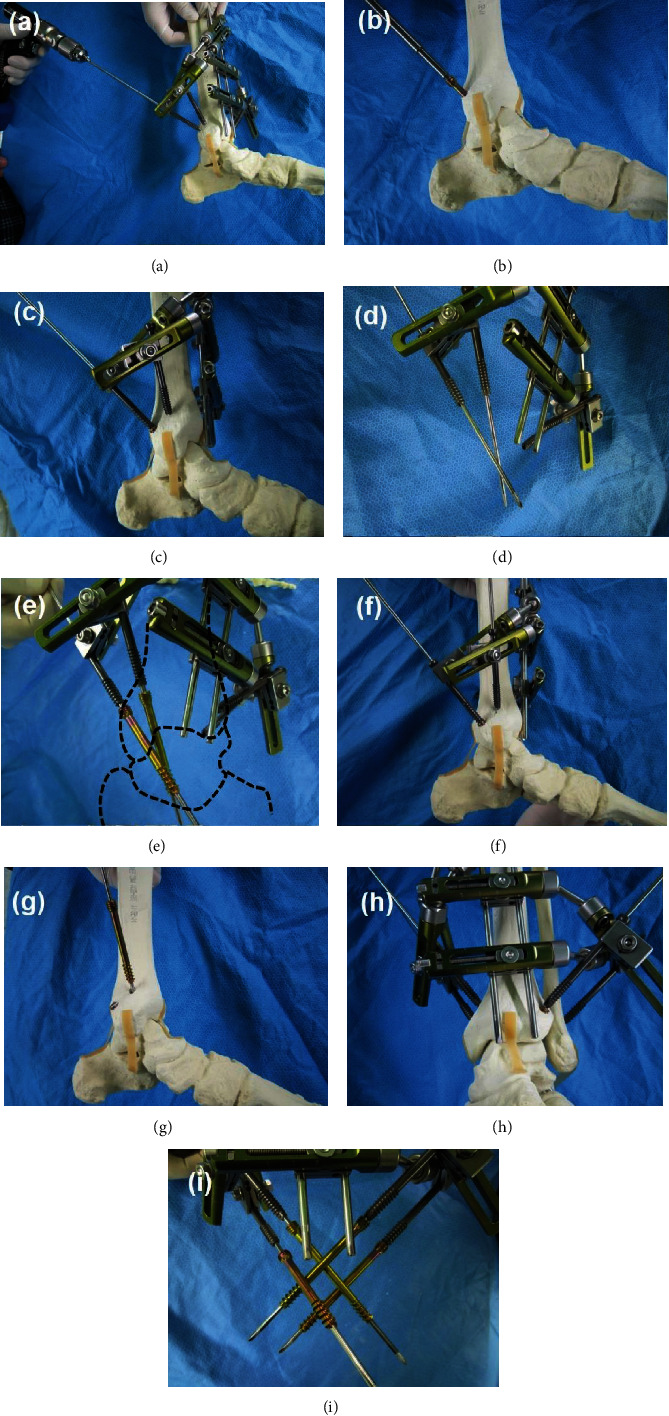
(a) A guide pin is inserted through the holes of the cannulated guide screw where it can be inserted percutaneously from the posteromedial tibia to the talus. (b) The device is removed from the ankle joint and a 6.5 cannulated screw is inserted through the guide pin. (c) The device is positioned again at the same place. Under C-arm fluoroscopic guidance, the second cannulated guide screw is adjusted for the second guide pin insertion. (d) Before inserting the second guide pin, the device is removed to check for the second screw trajectory. After removing the device from the ankle joint, guide pins are inserted through the two cannulated guide screws. (e) Two 6.5 cannulated screws are placed into the guide pins to simulate screw insertion inside the talus. When the collision is expected, the second screw trajectory can be adjusted. (f) The device is positioned on the ankle again using the same previous methods, and the second guide pin is inserted through the second cannulated guide screw which will not collide with the first screw. (g) The second 6.5 cannulated screw is inserted through the second guide pin. (h) With the same procedures, the device is positioned again and (i) the third and fourth screw trajectory can be simulated and can be inserted without collision. The dotted lines outline the bones around the ankle joint.

The patient is placed in the supine position. After the thorough removal of the articular cartilage and abrasion of the underlying subchondral bone surface by the arthroscopic technique, all the instruments and the arthroscope are removed to prepare for screw fixation. The 3-dimensional navigation drill guide is positioned on the ankle joint where the lower-end of the medial S-pin will sit on the medial corner of the ankle joint (Figure 3(b)). Under C-arm fluoroscopic guidance, a guide pin is inserted through the hole of the cannulated guide screw where it can be inserted percutaneously from the posteromedial tibia to the talus (Figure 4(a)). Joints of the external fixator can be loosened for adjustment. Once the guide pin is inserted, all the joints of the external fixator are locked and the device is removed from the ankle joint leaving the guide pin. Subsequently, a 6.5 cannulated screw is inserted through the guide pin (Figure 4(b)). The device is positioned again at the same place by putting the cannulated guide screw through the guide pin and the lower-end of the medial S-pin on the medial corner of the ankle joint. Under C-arm fluoroscopic guidance, the second cannulated guide screw is adjusted for the second guide pin insertion (Figure 4(c)). Before inserting the second guide pin, the device is removed to check for the second screw trajectory. After removing the device from the ankle joint, the guide pins are inserted through the two cannulated guide screws (Figure 4(d)) and two 6.5 cannulated screws are placed into the guide pins to simulate screw insertion inside the talus (Figure 4(e)).

**Figure 5:**
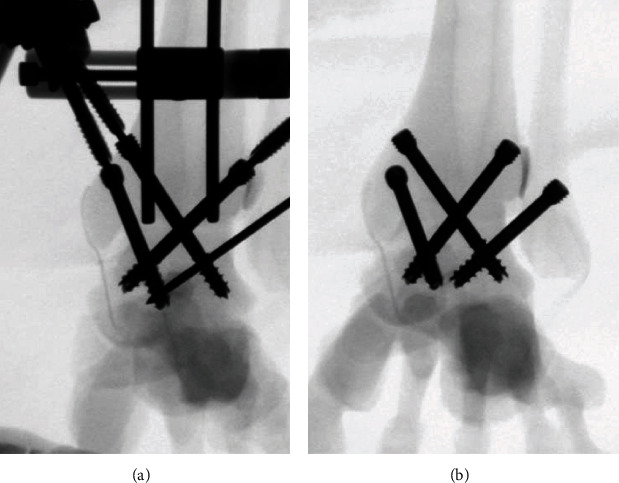
(a) C-arm fluoroscopic view on the sawbone model shows insertion of the fourth guide pin without any collision with screws using the 3-dimensional navigation device. (b) Four screws could be inserted without redrilling with the use of the device.

This simulation can show whether the two screws will collide inside the talus. When the collision is expected, the trajectory of the second screw can be adjusted. The device is positioned on the ankle again using the same method used previously, and the second guide pin is inserted through the second cannulated guide screw which will not collide with the first screw (Figure 4(f)). After removing the device, the second 6.5 cannulated screw can be inserted through the second guide pin (Figure 4(g)). The device is positioned again, and the third screw trajectory can be simulated before insertion of the 6.5 cannulated screw in a manner not to collide with the previously inserted two screws. With the same procedures, the fourth screw trajectory can be simulated and be inserted without collision (Figures 4(h), 4(i), and 5). This device can be sterilized and can be reused for arthroscopic ankle arthrodesis.

### 2.3. Implementation on Sawbone Models

Four orthopedic residents who had no prior experience of ankle arthrodesis were instructed by an experienced surgeon on how to use the 3-dimensional navigation drill guide and where to insert the screws for ankle arthrodesis. Each resident inserted 6.5 cannulated screws on 8 sawbone ankle models using the device and the C-arm fluoroscopy. The number of screws inserted without redrilling was recorded. When 5 screws could be inserted, it was regarded as successful and no further screws were inserted. An experienced attending surgeon also inserted the same screws on 2 sawbone ankle models to find out if there is any difference between the experienced and inexperienced surgeons.

## 3. Results

**Figure 6:**
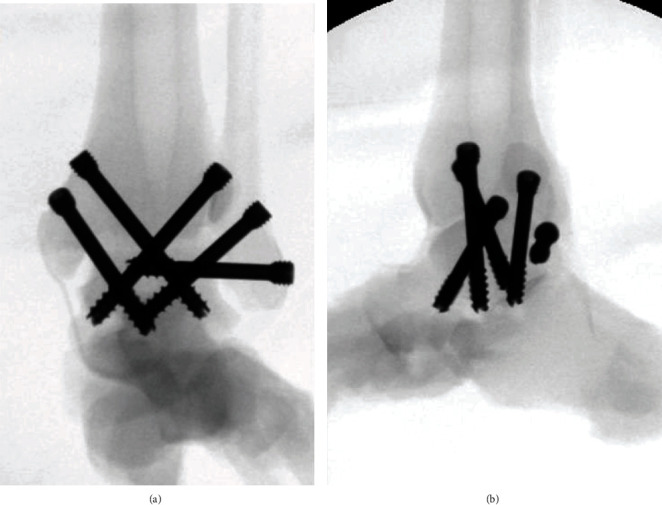
C-arm fluoroscopic (a) anteroposterior and (b) lateral views show insertion of 5 screws without collision.

All four residents and an attending surgeon did not experience any collision of screws for the three cannulated screws. Notably, one resident had collision of the 4th screw on his first sawbone model. However, the same collision on the second sawbone model did not occur (Table 1). On the second saw bon model, all surgeons could insert 5 screws without redrilling (Figure 6).

## 4. Discussion

The advantage of arthroscopic ankle arthrodesis over open arthrodesis is that it is minimal invasive and protects the periarticular blood supply to enhance the fusion process and facilitates faster repair. However, more rigid fixation methods such as plates cannot be implemented as it does not open the ankle joint [18, 19]. Constructing a more rigid stability with arthroscopic ankle arthrodesis will increase the fusion rate with rapid healing. Several reports show that a higher number of screws provide a more rigid fixation [14, 15, 17, 20]. In a finite element analysis, 3-screw fixation was predicted to provide higher initial stability over 2-screw fixation [15]. Ogilvie-Harris et al. [17] observed greater resistance to torque with a 3-screw configuration compared to 2-screw configuration, when measuring the gross motion between the tibia and talus in cadaveric specimens. Clinically, Yoshimura et al. [14] reported that 3 screws permitted faster time to union compared to 2 screws after studying 50 ankles with arthroscopic ankle arthrodesis. High union rates and function scores had been reported using 4 screws for ankle arthrodesis [20, 21]. However, compared to the open technique, inserting screws under C-arm fluoroscopic guidance for arthroscopic arthrodesis is difficult because the joint anatomy is not opened and visualized [22]. It is observed occasionally in open arthrodesis that the screws collide inside the bone delaying the operation time and increasing the radiation exposure. Computer-assisted navigation systems are developed to help surgeons to look into the bone and refine their procedures [23–27]. In a cadaveric study, accurate screw placement was possible in the narrow periacetabular bony corridor with the help of the electromagnetic navigation system [24]. The clinical applications of this computer navigation system are increasing [23, 26, 27]. However, not all surgeons have access to these new systems. Compared to these systems, our device is significantly cheaper as we can reuse the external fixators after their role to fix fractures. Once they are constructed, they can be reused after sterilization just like other operation tools.

**Figure 7:**
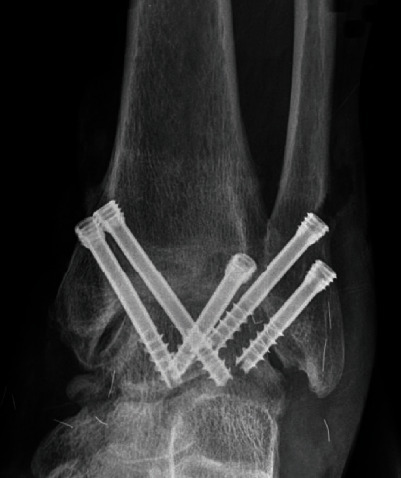
Five 6.5 screws could be inserted in patients using the 3-dimensional navigation drill guide.

Four novice surgeons who had no prior experience of ankle arthrodesis could insert 3 screws without redrilling using this device on their first trial sawbone model and could insert 5 screws into the second sawbone model. However, we acknowledge that the clinical setting in real patients is quite different from the sawbone model trial. When compared to the sawbone model trial, the patients are in a supine position. Moreover, it will not be easy to place the ankle in the best position for screw insertion. Also, anatomical landmarks are exposed in the sawbone models, which are not seen in the arthroscopic ankle arthrodesis. However, we could implement this device in the patients undergoing arthroscopic ankle arthrodesis with successful insertion of 5 screws (Figure 7). Further studies on the application of this device to more patients are required.

## 5. Conclusion

A 3-dimensional navigation drill guide constructed with external fixators can assist surgeons in implementing percutaneous screws for arthroscopic ankle arthrodesis.

## Figures and Tables

**Table 1:** Implementation of the 3-dimensional navigation drill guide on sawbone models.

	First sawbone model	Second sawbone model
Resident 1	4	5
Resident 2	5	5
Resident 3	5	5
Resident 4	5	5
Attending surgeon	5	5

Numbers of screws inserted without redrilling are presented.

## Data Availability

The datasets used and analyzed during the current study are available from the corresponding author on reasonable request.
